# Degradation and biological properties of Ca-P contained micro-arc oxidation self-sealing coating on pure magnesium for bone fixation

**DOI:** 10.1093/rb/rbu014

**Published:** 2014-12-13

**Authors:** Weidan Wang, Peng Wan, Chen Liu, Lili Tan, Weirong Li, Lugee Li, Ke Yang

**Affiliations:** ^1^Institute of Metal Research, Chinese Academy of Sciences, 72 Wenhua Road, Shenyang 110016, China, ^2^University of Chinese Academy of Sciences, Beijing 100049, China, ^3^EONTEC Co., Ltd, Dongguan, Guangdong, 523662, China

**Keywords:** pure magnesium, degradation, self-sealing, micro-arc oxidation coating, *in vivo* implantation

## Abstract

Poor corrosion resistance is one of the main disadvantages for biodegradable magnesium-based metals, especially applied for bone fixation, where there is a high demand of bio-mechanical strength and stability. Surface coating has been proved as an effective method to control the *in vivo* degradation. In this study a Ca-P self-sealing micro-arc oxidation (MAO) coating was studied to verify its efficacy and biological properties by *in vitro* and *in vivo* tests. It was found that the MAO coating could effectively retard the degradation according to immersion and electrochemical tests as well as 3D reconstruction by X-ray tomography after implantation. The MAO coating exhibited no toxicity and could stimulate the new bone formation. Therefore, the Ca-P self-sealing MAO coating could be a potential candidate for application of biodegradable Mg-based implant in bone fixations.

## Introduction

Currently, magnesium-based metals have gained increasing attentions for medical applications due to their unique characteristic of biodegradation in the physiological environment. It is worth that magnesium is essential to human metabolism, and the density, elastic modulus and yield strength of magnesium-based metals are much closer to the human bones than other metal implants, which can further reduce the stress shielding effect [1–3]. Besides, the biodegradation of magnesium implants *in vivo* will make the patients free from second operations for implant removals. However, the rapid corrosion is the major impediment in using them as orthopedic implants, especially for bone fixation due to lack of biomechanical strength and stability [4].

Surface treatment/coating is a viable approach to reduce the corrosion rate of magnesium-based metals and also provides a suitable surface for better bone bonding and cell growth. Some surface modification techniques have been developed in the past years, such as chemical deposition [5], micro-arc oxidation [6–8], electrodeposition [9], biomimetic method [10], dip-coating [11–13], Sol-gel [14], etc. Micro-arc oxidation (MAO) is a common technique for corrosion protection of magnesium alloys in the industrial sector [6]. By the MAO process, a relatively thick, dense and hard oxide coating can be produced on the surface of magnesium alloys [15]. Taking into account the similar chemical composition to bone, calcium phosphate-based electrolytes have been widely used to obtain bioactive MAO coating which could slow down the corrosion rate and also increase the biocompatibility [16]. Yao *et al*. [17] introduced Ca and P into a ceramic coating on a MAO-treated AZ91D alloy and found the coating reduced the corrosion current density by two orders of magnitude. Gan *et al*. [7] prepared a calcium phosphate coating on pure magnesium, and found that the calcium phosphate could deposit in the micropores of MAO coating, which could effectively decrease the degradation rate in the *in vitro* immersion test. Overall, the coating should provide magnesium-based metals a controlled degradation rate and also better surface bioactivity, which promotes the early bone growth at the implant/bone interface for orthopedic application.

Based on our previous study [7], a Ca-P self-sealing coating was fabricated by MAO technique. To evaluate the preclinical efficacy of the coating for biodegradable bone fixations made of magnesium-based metal, the *in vitro* and *in vivo* degradation properties as well as the biological properties were systematically studied by means of scanning electron microscopy (SEM), energy-dispersive spectroscopy (EDS), electrochemical test, *in vitro* immersion, cytotoxicity evaluation and *in vivo* implantation using X-ray tomography (XRT).

## Experimental

### Preparation and characterization of MAO coating

Samples (Ф10 mm × 5 mm) of pure magnesium (99.99%, EONTEC Co., Ltd, Dong Guan, China) were used as the substrate for MAO coatings. The samples were ground and polished successively with 600, 800, 1200, 2000 grit emery sheets before the MAO treatment. The based electrolyte was prepared from the solution of calcium hydroxide (0.8 g/l), sodium hexametaphosphate (3.5 g/l) and potassium fluoride (8 g/l) in distilled water. The MAO process was implemented at a constant working voltage of 360 V for 5 min. All coated samples were rinsed thoroughly in distilled water and dried in ambient air immediately after the MAO treatment.

The surface and cross-section morphologies of the MAO coatings were identified on a scanning electron microscopy (SEM, S-3400N, Hitachi, Japan), equipped with an EDS system.

### Electrochemical measurements

Electrochemical tests were carried out using an electrochemical workstation (Verserstat3, Princeton Applied Research, USA) in the Hank’s balanced salt solution (HBSS 10-527, Lonza Walkersville, USA) at 37 ± 0.5°C by using a conventional three electrodes system with a saturated calomel electrode (SCE) as the reference, a platinum electrode as the counter and the sample as the working electrode. The samples were allowed to stabilize at their open circuit potentials (OCP) for 30 min before the measurements were started. Potentiodynamic polarization was measured at a sweep rate of 0.5 mV/s within a scan range of ± 0.25 V with reference to OCP. The electrochemical tests were conducted in triplicate in order to ensure the reproducibility of results.

### *In vitro* immersion tests

The samples were immersed in the Hank’s solution at 37 ± 0.5°C for 28 days. The ratio of surface area/solution volume was 1.25 cm^2^/ml. The immersion solution was replaced every 24 h, with HCl and NaOH solution adjusting pH to about 7.4. The pH value of the solution was measured at 24 h intervals during the immersion test. The weight loss was measured after 28 days to calculate the corrosion rate according to ASTM G31-72. The corrosion products were cleaned using chromic acid solution (200 g/l CrO_3_ and 10 g/l AgNO_3_) for 15 min in an ultrasonic bath at room temperature until all the corrosion products and coating were removed.

### Cytotoxicity

The cytotoxicity test was carried out with L929 cells by extract assay according to ISO 10993-5. Extracts were prepared with a ratio of surface area over extraction medium of 1.25 cm^2^/ml in a humidified atmosphere containing 5% CO_2_ at 37°C for 24 h incubation.

Cells were incubated in 96-well plates at 2 × 10^4^ cells per 100 µl in each well and incubated for 24 h to allow attachment. The medium was then replaced with 100 µl of the extract, 100 µl of the negative control (medium alone) or 100 µl of the positive control (10% Dimethyl sulfoxide (DMSO) medium). After incubation for 1, 3 and 5 days, respectively, 10 µl of 3-(4,5-dimethyl-2-thiazolyl)-2,5-diphenyl-2-H-tetrazolium bromide (MTT) was added to each well for 4 h and 100 µl of formazan solubilizing solution (10% Sodium dodecyl sulfate (SDS) in 0.01 M HCl) was added to each well overnight. The spectrophotometric absorbance of each well was measured using a microplate reader (Bio-Rad) at 490 nm with a reference wavelength of 570 nm [18]. All the above experiments were conducted in triplicate. A cell viability ratio (CVR) for every sample was calculated to evaluate the cytotoxicity using the equation: CVR = (viable cell count in experimental extract)/(viable cell count in control extract), according to ISO10993-5.

### *In vivo* implantation study

#### Surgery

Ethical approval was obtained for the animal tests that were conducted according to the ISO 10993-2 animal welfare requirements. Eight 4-month-old male New Zealand white rabbits were used in this study, randomly assigned to two groups (*n* = 4). All rabbits were generally anesthetized with ketamine (75 mg/kg) and xylazine (10 mg/kg) for surgery and the left knee scrubbed with 25 g/l tincture of iodine and 70% ethanol. For the experimental group the MAO-coated magnesium rods (Ф2 × 6 mm) were implanted into predrilled bone tunnels in the femur along the axis of the shaft from the distal femur using established surgical and model protocols [19]. In the control group the predrilled bone tunnel was filled with pure magnesium rods for comparison. The wounds were then carefully sutured and the mice were housed in an environmentally controlled animal care laboratory after surgery.

#### XRT (micro-CT) evaluation

An *in vivo* high resolution transmission X-ray tomography (HRTXRT) set-up (VersaXRM-500,) with a voxel size of 1 µm was used to monitor the distal femora of rabbit at the implantation periods of 4, 8 and 12 weeks post-surgery. Two-dimensional images were acquired directly from the scans and the three dimensional structure was reconstructed using the volume of interest, with an optimized threshold used to isolate the bone and materials from the background. The volume changes of the MAO coating and pure magnesium implants of the digitally extracted tissue were measured. The *in vivo* degradation rate was calculated according to the equation [20]:

$$C=\left(V_{0}-V_{t}\right)/At$$

where *C* is the corrosion rate, *V_t_* is the sample volume measured by XRT at different implantation intervals, *V_0_* is the sample volume measured by XRT on week 0, *A* is the initial implant surface area, and *t* is the implantation time based on our established protocol [21].

#### Histological evaluations

After the rabbits were killed 4, 8 and 12 weeks post-surgery, the femora were harvested and fixed in 10% buffered formalin. After gradient dehydration, the harvested femora were embedded and polymerized in methylmethacrylate resin. Uncalcified sections of 20 µm thickness were prepared using Leica RM2235 rotary microtome instruction, perpendicular to the long axis of the femoral shaft. The cross-sections were stained with Stevenel’s blue and Van Gieson’s picro fuchsin and subsequently observed under optical microscopy.

#### SEM characterization

For degradation product analysis after implantation, tissue blocks containing the implants were fixed in a 2.5% glutaraldehyde solution for 24 h, gradient alcohol dehydrated and embedded in the epoxy resin. The resin blocks with bone tissues were ground perpendicular to the long axis of the implant to get cross-sections of the implants with the surrounding bone tissues. Then the bone- implant interfaces were characterized on SEM and the chemical composition was determined by EDS.

### Statistical analysis

Statistical analysis was conducted with SP13.0. Differences among groups were analyzed using Multi-ANOVA followed by Tukey’s test. *P* ≤ 0.05 was set as statistical difference.

## Results and Discussion

### Morphologies and chemical compositions of the MAO coatings

**Figure 1:**
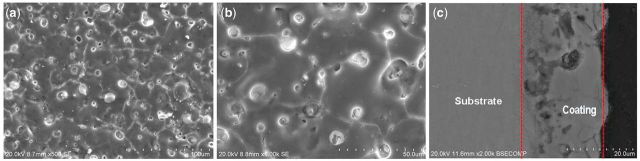
Surface (**a, b**) and cross-sectional (**c**) morphologies of MAO coatings on samples.

The surface and cross-sectional morphologies of the MAO coatings were shown in Fig. 1. It was found that the morphologies of the coatings were different from the typical MAO coating feature with porous structure. Obviously, most of the micro-size pores were filled with compound particles. These particulate substances sealed in the micro-pores were mainly contributed to the simultaneous deposition from Ca-P containing electrolyte during MAO process. As to the cross-section morphologies shown in Fig. 1c, the self-sealing MAO coatings show strong integration with the magnesium substrate and are relatively compact and uniform with fewer defects in the inner regions of the coating. The average thickness of the coatings is ∼20 µm.

**Figure 2:**
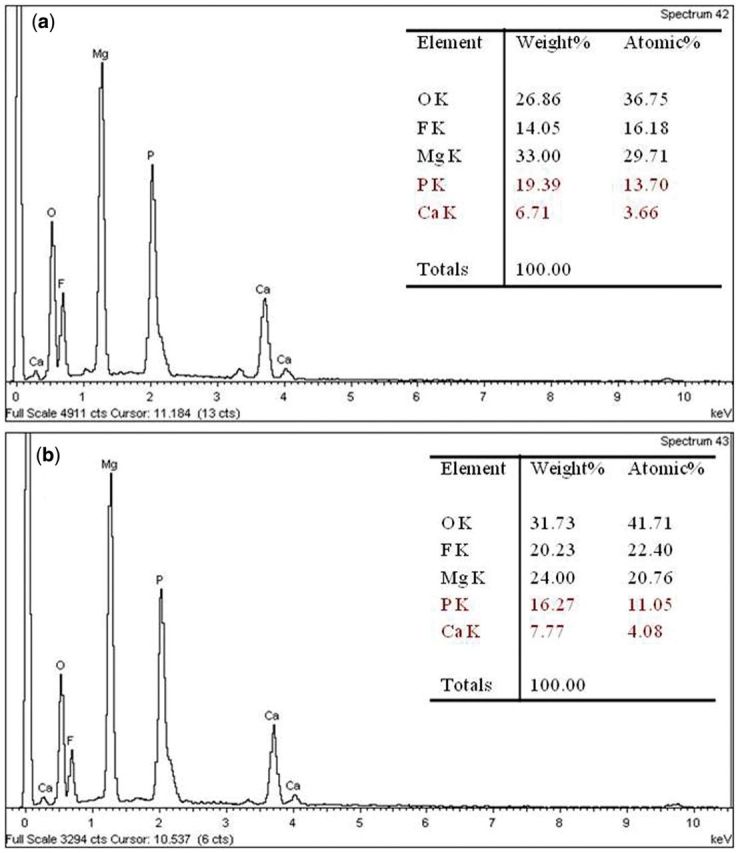
EDS point-scan analysis of Ca-P-containing MAO coatings on samples; (**a**) coating surface, (**b**) filler in the micro-pores.

The EDS analysis on the MAO coatings obtained was conducted and is shown in Fig. 2a and b, which represent the elemental concentrations in the position of coating surface and compound particles filling in the micro-pores, respectively. Mg, O, F, P and Ca are the major elements identified in the coatings. Obviously, Mg, O and F participated in the film formation, indicating that the coating was mainly composed of MgO. Ca and P came from the electrolytes, which might be owing to the diffusion and electrophoresis reaction that manifested both the magnesium substrate and electrolyte components involved in the coating formation during the MAO process. Furthermore, the Ca content in the compound particles is significantly higher than that in the coating. Thus, it can be deduced that the filled compound particles are mainly composed of Ca-P deposits, whereas the coating may contain more magnesia and magnesium phosphate.

### Electrochemical behaviors

**Figure 3:**
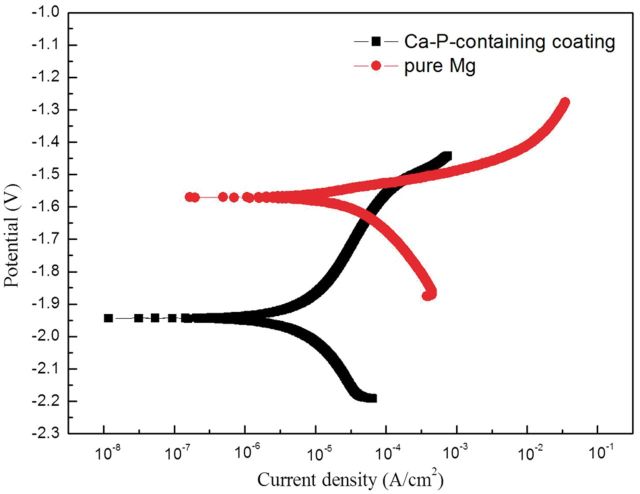
Polarization curves of pure Mg with and without MAO coating.

Figure 3 exhibits the potentiodynamic polarization curves of pure Mg and MAO coating samples in HBSS and the corresponding electrochemical fitting data are shown in Table 1. The anodic polarization curve represents the dissolution of magnesium, whereas the cathodic one represents the hydrogen evolution through the reaction. As can be seen from the polarization curves, the corrosion current density in the anode region of the pure magnesium substrate increases sharply, meaning that severe corrosion occurred on pure magnesium in Hank’s solution, whereas for MAO coating samples both the cathodic and anodic corrosion current densities are significantly lower than those of pure Mg, representing that the metal dissolution process should be inhibited. The corrosion current density of the MAO coating rises slowly from corrosion potential (*E*_corr_) to ∼−1.5 V versus SCE, then increases rapidly, meaning that the MAO coating could effectively inhibit the corrosion of pure Mg when potential was <−1.5 V. The current density by Tafel fitting and the calculated corrosion rate (CR) according to the ASTM-G102-89 standard of samples are listed in Table 1. Both *I*_corr_ and CR for MAO coating samples are one order lower than those of the pure Mg, which indicates that the MAO coating could effectively inhibit the occurrence of corrosion.

**Table 1:** Corrosion rate, current and polarization resistance of samples fitted from polarization curves

Samples	Ecorr (V)	Icorr (µA/cm^2^)	CR (mm/year)
Pure Mg	−1.80	32.0	0.17 ± 0.014
Ca-P containing coating	−1.91	8.87	0.049 ± 0.002

### *In vitro* immersion behaviors

**Figure 4:**
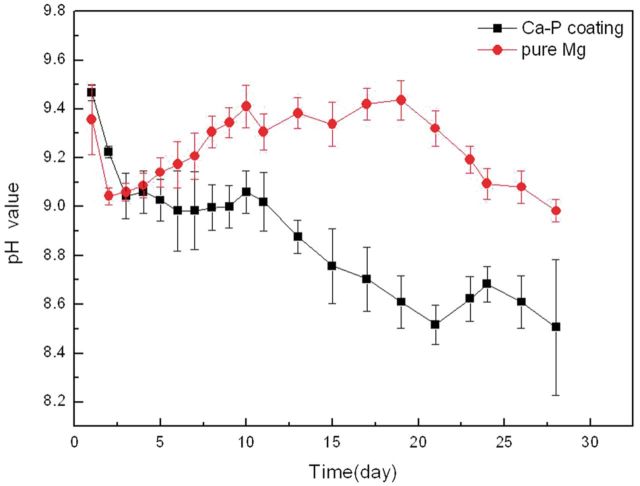
pH variations of Hank’s solution immersed with pure Mg with and without MAO coating.

Electrochemical test results showed that MAO coating could significantly improve corrosion resistance of pure Mg in Hank’s solution. However, what it reflected was the initial immersion stage when the coating had a good protection of pure Mg substrate. Therefore, *in vitro* immersion tests were conducted to evaluate the long-time degradation behavior of the samples. Figure 4 shows the pH variation of Hank’s solution immersed with pure Mg with and without MAO coating for up to 28 days. During the initial immersion stage, pH for pure Mg reached a maximum value of about 9.5, meanwhile, pH for the MAO-coated Mg approached to the maximum value with even slightly higher than that of pure Mg in the first 3 days. On the subsequent immersion stage, pH for MAO coating gradually decreased, and significantly showed a lower value than that of pure Mg. With the extension of immersion time, a fluctuated change of pH for both samples occurred. However, all the pH curves presented a downward trend overall.

The higher pH for the MAO-coated sample was shown at the initial immersion time due to the presence of pores, defects, corrosive liquids on the sample surface, on the one hand, which could penetrate through these channels to the magnesium substrate, causing localized corrosion and solution basified. On the other hand, the MgO phase contained in the coating was also involved with the degradation process because of its poor chemical stability. When contacted with an aqueous solution, MgO would hydrate, producing a more stable Mg(OH)_2_ phase, and then released more OH^−^ by reaction with Cl^−^ in the Hank’s solution.

For the fluctuations of pH changes appeared during the immersion process, owing to a layer of corrosion products comprising MgO, Mg(OH)_2_ would be deposited on the surface of samples, resulting in a decrease of Mg ions concentration in the solution. Meanwhile, the deposition of corrosion products was relatively loose, providing a weak protective effect on the pure Mg substrate. As a result, with the deposition and dissolution of surface corrosion products, especially Mg(OH)_2_, pH of the extract showed a jagged fluctuation. In comparison, the MAO-coated sample showed certain inhibition due to the corrosion product deposition of both phosphate and carbonate of calcium and magnesium on the sample surface. Consequently, the Ca-P contained MAO coating indicated a better resistance of corrosion in HBSS.

**Figure 5:**
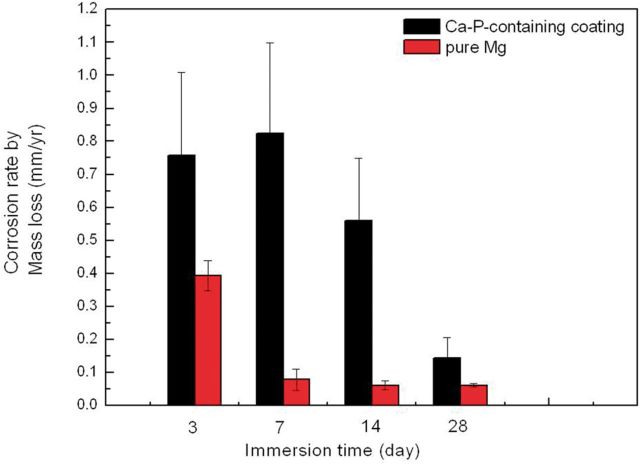
Corrosion rate by mass loss of pure Mg with and without MAO coating during different immersion time.

Figure 5 shows the corrosion rate by mass loss of pure Mg with and without MAO coating during different immersion times in HBSS, referring to the formula as below.

$$CR=\frac{KW}{ATD}$$

where, *CR* is the corrosion rate (mm/year), *K* is the constant of 8.76 × 10^4^, *T* is the immersion time (accurate to 0.01 h), *A* is the sample surface area (accurate to 0.01 cm^2^), *W* is the mass loss (accurate to 1 mg) and *D* is the density (g/cm^3^).

With the extension of time, degradation rate of pure Mg with and without MAO coating all declined. However, the MAO-coated sample showed faster degradation rate than that of pure Mg, which may be caused by the weight loss from the film formation process during the MAO reaction, consuming partial Mg substrate into the MgO in the coating. Therefore, the actual original weight of the pure Mg substrate reduced, leading to inaccurate conclusion in the subsequent evaluation results for mass loss. Based on this, mass loss might not be suitable to measure the degradation rate for MAO-coated samples.

### Cytotoxicity

**Figure 6:**
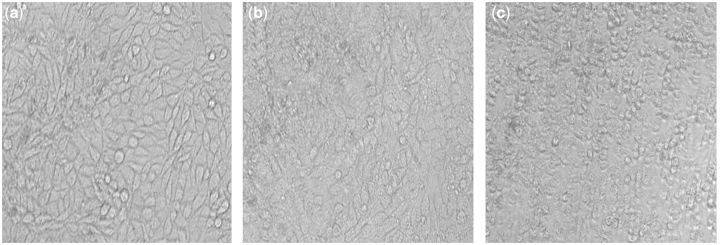
Cell morphologies in the MAO coating group (**a**), blank group (**b**) and positive group (**c**) after cultured with L929 for 24 h.

**Figure 7:**
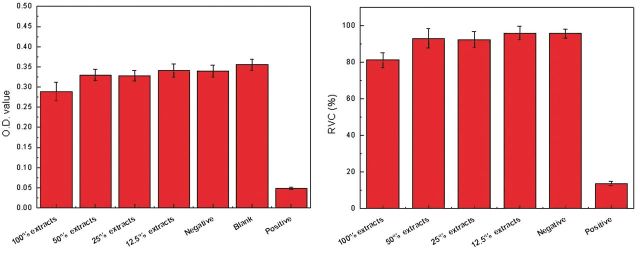
OD value and cell viability results for MAO coating samples by MTT test.

*In vitro* cytotoxicity of the Ca-P containing MAO-coated samples was assessed by measuring the viability of the L929 cells after 24 h of culture in the extraction mediums according to ISO10993-5, with blank group and DMSO as the controls. Figure 6 depicts the growth morphologies of L929 cells incubated with the extract of MAO-coated sample for 24 h. Large amount of natural fusiform L929 cells could be found in the MAO coating group compared with blank group, indicating that the coating did not inhibit the cells growth and proliferation. On the contrary, the DMSO group showed severe cytotoxicity. The optical density (OD) value and cell viability by MTT test are shown in Figure 7. It can be seen that no cytotoxicity for all the extracts of MAO-coated samples was found, and the cell viabilities of coating are all above 80% and close to that of blank group, indicating that coating could significantly retard corrosion and thus display excellent biocompatibility. With increase in the dilution ratio of extract, the coating led to the highest cell viability and showed cytotoxicity of grade 0. Cytotoxicity of grade 0–1 was shown by the Ca-P MAO-coated sample according to ISO10993-5, suggesting good cytocompatibility and acceptable biosafety for cellular applications.

### *In vivo* degradation by XRT

XRT is a technology that uses computer-processed X-rays to produce tomographic images of specific areas of the scanned object, allowing the user to see what is inside of it without cutting it open. Medical imaging is the most common application of X-ray CT. Its cross-sectional images are used for diagnostic and therapeutic purposes in various medical disciplines [22]. It was found that the *in vitro* degradation measurements including hydrogen evolution, mass loss and electrochemical behavior could not factually reflect the *in vivo* status [23], thus *in vivo* degradation after implantation is needed for non-destructive examination in 3D by XRT.

**Figure 8:**
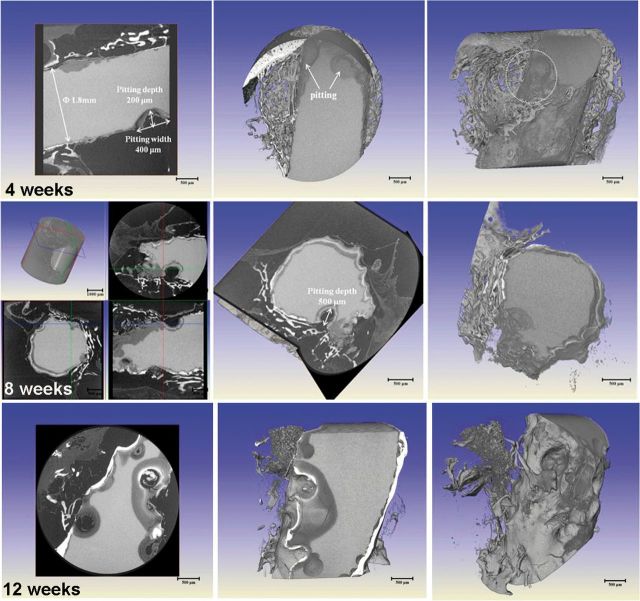
2D slice and 3D reconstruction morphologies of pure Mg at implantation periods of 4, 8 and 12 weeks by HRTXRT.

Figure 8 shows the 2D slice and 3D reconstruction morphologies of presently studied pure Mg at implantation periods of 4, 8 and 12 weeks by XRT. It can be found that the localized corrosion attack occurred on pure Mg from the initial stage of 4 weeks. Corrosion occurred as irregular shallow or deep pits that were locally spread over the whole implant. The pits initialed on the edge and gradually spread to the core. The large pit was measured with size of 400 µm in width and 200 µm in depth. Furthermore, it was observed that the areas covered with bone tissue exhibited no or less local corrosion attack. On one hand, the bone wrapping could act as a protective layer for Mg implants, and on the other hand, the initial rapid corrosion could also influence the bone integration with the implant. With the extension of time, the pitting of pure Mg became severe and the implanted rod has lost the original shape and integrity. Till the implantation time of 12 weeks, the pits expanded and were connected throughout the Mg rod, and the pitting influence zone could reach half of the rest volume. It could be assumed that the implants at this stage could not give the mechanical support and lose efficacy for bone fixation.

**Figure 9:**
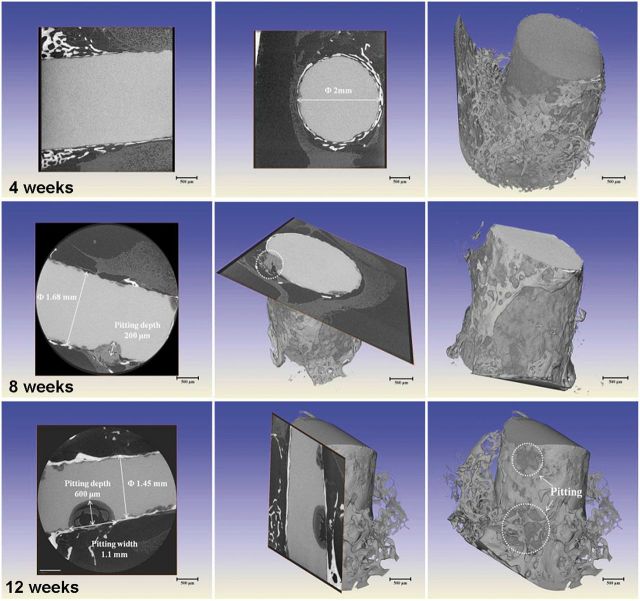
2D slice and 3D reconstruction morphologies of the MAO-coated sample at implantation periods of 4, 8 and 12 weeks by HRTXRT.

Comparatively, the Mg rod with MAO coating exhibited significant discrepancy as shown in Fig. 9. It can be clearly seen that during implantation of 4 weeks, the MAO-coated sample showed no corrosion tendency and kept an intact feature. It was proved that the coating could supply effective protection for pure Mg substrate at the initial period. With increase of time to 8 weeks, the diameter of rod decreased from 2 mm to 1.68 mm due to homogeneous corrosion and meanwhile occasional pit occurred on the edge of the sample. The MAO-coated sample still kept the integrated shape. However, once the pitting occurred, it rapidly spread and extended into the core. It could be observed that the large pit was ∼1.1 mm in width and 600 µm in depth at implantation of 12 weeks. Thus, it can be deduced that the MAO coating could supply a corrosion protection and keep the mechanical integrity for at least 3 months. Measurements of the residual implant volume indicate the pure magnesium sample degraded by 19% of its initial volume after 12 weeks post-surgery, whereas the MAO coating sample lost about 7% of its initial volume. According to the calculation formula mentioned above, the corrosion rates of pure Mg after 4, 8 and 12 weeks’ implantation are 2.24, 1.75 and 1.28 mm/yr, respectively; whereas those of MAO coating after 4, 8 and 12 weeks’ implantation are significantly decreased to 0.311, 0.28 and 0.566 mm/yr, respectively. The MAO-coated Mg degrades significantly slower than the uncoated pure magnesium.

### SEM characterization and EDS analysis

**Figure 10:**
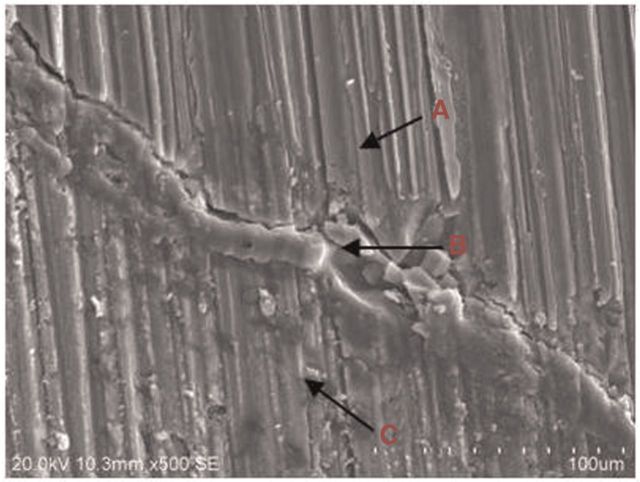
SEM image of the MAO-coated pure Mg implant and the surrounding bone tissues after 4 weeks’ implantation.

Figure 10 shows the typical cross-section images of the MAO-coated sample at implantation of 4 weeks. It can be clearly seen that the MAO coating is still present, and the interface between coating and the surrounding bone tissue shows tight combination without gap. EDS analysis on three sites listed in Table 2 gives the elemental composition. The intermediate part near Mg substrate is considered as the degraded coating and deposited bone mineral, which is reflected by amounts of Ca and P distributions. The far end of position C with Ca/P ratio of 1.62 is proved to be adhered on the bone tissue. Considerable new bone tissues were formed around the MAO-coated rod at week 4, probably due to the proper release of magnesium ions [24].

**Table 2:** elemental composition of each region in Fig.10 by EDS analysis

Position	Elements(at%)				
	C	O	Mg	P	Ca
A	0	0	100	0	0
B	54.00	27.54	6.73	5.06	6.67
C	33.95	40.04	1.33	9.44	15.24

**Figure 11:**
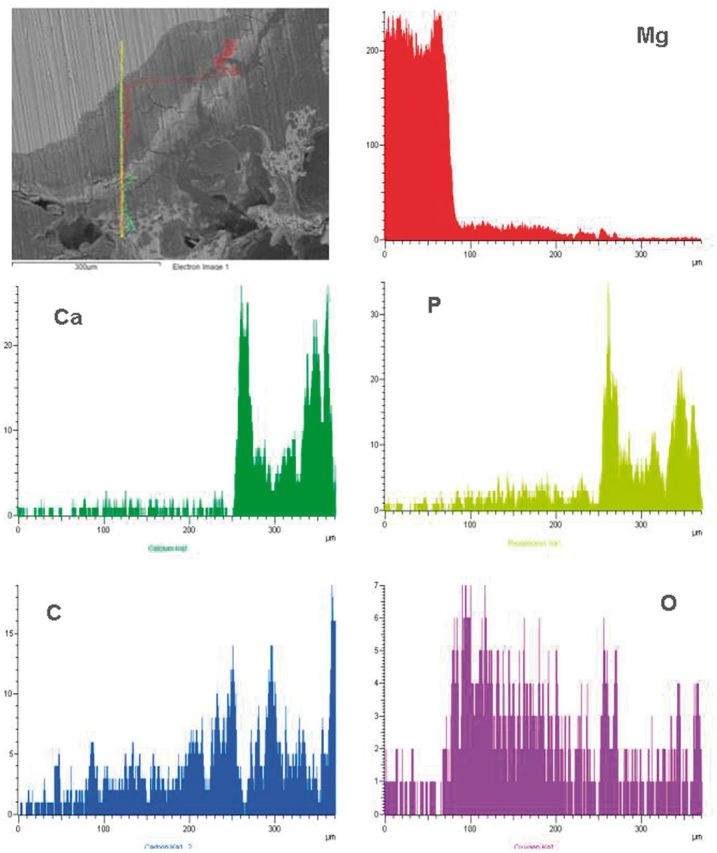
element lining analysis on an interface of the MAO-coated Mg-bone at 4 weeks post-surgery.

**Figure 12:**
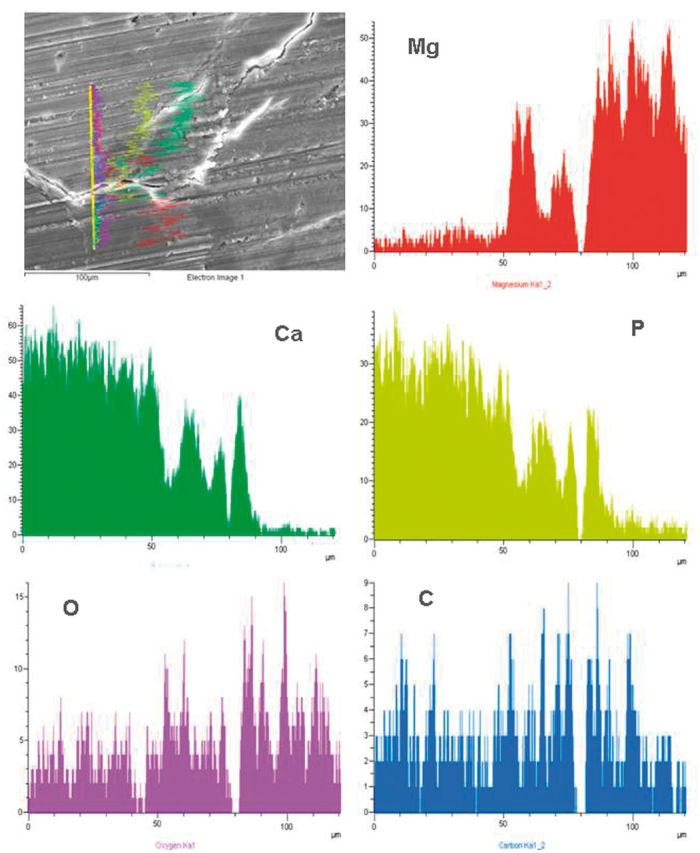
element lining analysis on an interface of the MAO-coated Mg-bone at 12 weeks post-surgery.

The line scan from substrate to bone was adopted to analyze the degradation of coating at weeks 4 and 12 post-surgery according to the elements distribution, as shown in Figs 11 and 12. There is sudden increase of O content near the inner Mg substrate, which is corresponding to the main corrosion products. The existence of MAO coating outward is proved with increase of Ca, P and O, meanwhile including bone-like calcium phosphate deposition. The thickness for the rest of coating is about 10 µm. The outer layer with high Ca and relatively low P contents is indicated as wrapped bone tissue. It can be deduced that the inner Mg substrate was corroded along with simultaneous coating degradation. The body fluid penetrated into the Mg substrate through micro-pores in the coating, resulting in its corrosion, meanwhile the weak adhesion between coating and substrate also induced degradation and segregation of coating layer. At week 12 post-surgery, the similar linear element distribution was observed with that at week 4. However, the thickness of MAO coating was significantly reduced to 5 µm, indicating the continuous degradation of coating with time. Meanwhile, there were more bone-like mineral deposition and new bone growth at week 12 post-surgery, which was reflected by increase of Ca and P contents.

### Histological analysis

**Figure 13:**
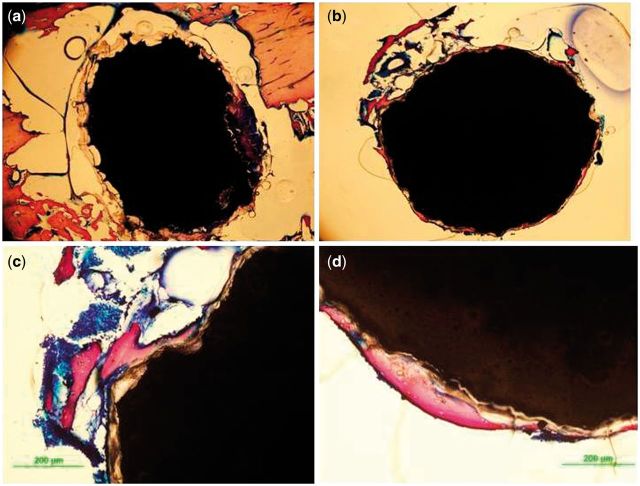
histological thin slides of pure Mg (**a**) and MAO-coated Mg (**b, c, and d**) implants at implantation of 4 weeks.

**Figure 14:**
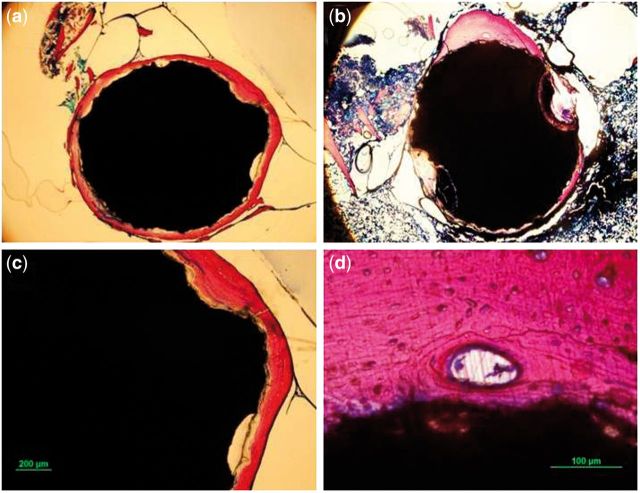
histological thin slides of pure Mg (**a, c**) and MAO-coated Mg (**b, d**) implants at implantation of 12 weeks.

Figures 13 and 14 show the histology of cross-sections of the bone-implanted sample. The implanted MAO-coated rod maintained its central integrity, whereas the pure Mg was observed with pits around the edge of rod. Due to the initial rapid corrosion, the corroded products, which were stained in brown yellow color, were accumulated and wrapped outside of the Mg substrate without new-born bone. Comparatively, more bone trabeculae stained as purple could be seen for the MAO-coated sample. The compact layer of newly formed bone tissues closely contacted to the surface of MAO-coated rod with mild degradation (Fig. 13b). Figure 13d also displays that some of the new bone grew directly on the surface of MAO-coated sample, indicating good osteo-compatibility of the MAO coating as demonstrated *in vitro* [25].

It was interestingly found that amounts of newly formed bone tissues wrapped the pure Mg rod at week 12 post-surgery (Fig. 14a). It can be deduced that the more bone trabeculae formation was contributed to the slow corrosion rate and induced by stimulation of Mg ions. As to the MAO-coated sample, the obvious pitting occurred and spread into the core, which was consistent with the XRT result mentioned above. The MAO coating showed strong periosteal bone formation, which was reflected by the large amounts of osteoblasts and bone resorption lacuna (Fig. 14d). Both the groups exhibited bone formation adjacent to the former implant interface, producing comparatively unstructured woven bones and indicating the active bone remodeling at this time.

## Conclusions

The results of this study found that the Ca-P containing self-sealing MAO coating could effectively control the *in vitro* degradation of pure Mg according to the electrochemical and immersion tests. Additionally the mass loss for degradation evaluation was not suitable for the MAO-coated Mg because the Mg matrix was partly transformed into MgO coating during MAO process. No cytotoxicity was found for MAO coating, indicating that the MAO-coated pure Mg could satisfy the demand for medical device application. The *in vivo* implantation showed that pure Mg suffered from severe corrosion after 4-week implantation mainly in the form of pitting. Whereas, the MAO coating could significantly delay the rate of corrosion attack till 12 weeks and enhance the primary new formation of bone around the implant. In general, the Ca-P self-sealed MAO coating could be a potential candidate for application of biodegradable Mg-based implants in bone fixations.
